# The maxillary incisor display at rest: analysis of the underlying components

**DOI:** 10.1590/2177-6709.23.6.048-055.oar

**Published:** 2018

**Authors:** Waqar Jeelani, Mubassar Fida, Attiya Shaikh

**Affiliations:** 1 The Aga Khan University, Department of Surgery, Section of Dentistry (Karachi, Pakistan).

**Keywords:** Esthetics, Incisal display, Lip.

## Abstract

**Introduction:**

Maxillary incisal display is one of the most important attributes of smile esthetics.

**Objective:**

The aim of this study was to determine the relationship between maxillary incisal display at rest (MIDR) and various soft tissue, hard tissue and dental components.

**Methods:**

A cross-sectional study was conducted on 150 subjects (75 males, 75 females) aged 18-30 years. The MIDR was recorded from the pretreatment orthodontic records. The following parameters were assessed on lateral cephalograms: ANB angle, mandibular plane angle, palatal plane angle, lower anterior and total anterior facial heights, upper incisor inclination, upper anterior dentoalveolar height, and upper lip length, thickness and protrusion. The relationship between MIDR and various skeletal, dental and soft tissue components was assessed using linear regression analyses.

**Results:**

The mean MIDR was significantly greater in females than males (p = 0.011). A significant positive correlation was found between MIDR and ANB angle, mandibular plane angle and lower anterior facial height. A significant negative correlation was found between MIDR and upper lip length and thickness. Linear regression analysis showed that upper lip length was the strongest predictor of MIDR, explaining 29.7% of variance in MIDR. A multiple linear regression model based on mandibular plane angle, lower anterior facial height, upper lip length and upper lip thickness explained about 63.4% of variance in MIDR.

**Conclusions:**

Incisal display at rest was generally greater in females than males. Multiple factors play a role in determining MIDR, nevertheless upper lip length was found to be the strongest predictor of variations in MIDR.

## RESUMO

**Introdução:**

a exposição dos incisivos superiores é um dos fatores mais importantes na estética do sorriso.

**Objetivo:**

o objetivo desse estudo foi determinar a relação entre a exposição dos incisivos superiores em repouso (EISR) e diferentes componentes em tecidos moles, tecidos duros e dentários.

**Métodos:**

um estudo transversal foi conduzido com 150 pacientes (75 homens, 75 mulheres), com idades entre 18 e 30 anos. A EISR foi obtida nos registros pré-tratamento ortodôntico. Os seguintes parâmetros foram analisados nas radiografias laterais: ângulo ANB; ângulo do plano mandibular; ângulo do plano palatino; altura facial anterior inferior; altura facial anterior total; inclinação dos incisivos superiores; altura dentoalveolar anterior superior; comprimento, espessura e protrusão do lábio superior. A relação entre a EISR e os diferentes componentes esqueléticos, dentários e em tecidos moles foi analisada por meio de uma análise de regressão linear.

**Resultados:**

a EISR média foi significativamente maior nas mulheres do que nos homens (*p*= 0,011). Foi encontrada correlação positiva significativa entre a EISR e: o ângulo ANB, ângulo do plano mandibular e a altura facial anterior inferior. Uma correlação negativa significativa foi encontrada entre a EISR e o comprimento e a espessura do lábio superior. A análise de regressão linear mostrou que o comprimento do lábio superior foi o maior preditor da EISR, explicando 29,7% das variações na exposição dos incisivos superiores em repouso. Um modelo de regressão linear múltipla baseado no ângulo do plano mandibular, na altura facial anterior inferior, comprimento do lábio superior e espessura do lábio superior explicou cerca de 63,4% das variações na EISR.

**Conclusões:**

a exposição dos incisivos em repouso foi, de um modo geral, maior nas mulheres do que nos homens. Múltiplos fatores exercem influência na quantidade da exposição dos incisivos superiores em repouso; porém, o comprimento do lábio superior foi identificado como o maior preditor das variações na EISR.

## INTRODUCTION

Smile is one of the most important expressions contributing to the facial attractiveness. An attractive and pleasing smile enhances the acceptance of an individual in the society by improving interpersonal relationships.^1^ With patients becoming increasingly conscious of their dental appearance, smile esthetics has become the primary objective of orthodontic treatment.^2^ The most important esthetic goal in orthodontics is to achieve a balanced smile, which can be best described as an appropriate positioning of teeth and gingival scaffold within the dynamic display zone.^3^ A significant portion of maxillary incisors is also visible during speech, mastication and various facial expressions. The vertical exposure of the maxillary incisors during function is strongly correlated to the maxillary incisor display at rest (MIDR). 

Various studies have shown that people with pleasing smile esthetics have a MIDR ranging from 2 to 4 mm.^4,5^ Excessive exposure of the maxillary incisors at rest may result in gummy smile; whereas, the reduced incisor exposure is less esthetic and is considered a sign of aging.^4,5^ A significant proportion of orthodontic patients present to the dental clinics with the chief complaint of an excessive or reduced maxillary incisor display.^6^ The treatment planning for each patient aims at the correction of one or more hard or soft tissue components responsible for a less ideal incisal display. 

Several hard and soft tissue structures that surround and support maxillary incisors have been shown to affect the MIDR.^6^ ^-^ ^8^ An increased or reduced vertical skull dimensions and a discrepancy in the sagittal jaw relationship are the primary skeletal components affecting the MIDR. However, some authors also claim that the vertical maxillary excess (VME) is the strongest determinant of the maxillary incisor display.^9-11^ The height of anterior portion of maxilla is dependent on the dentoalveolar segment, as patients with extruded anterior teeth have greater anterior maxillary dentoalveolar height. Depending on the severity of VME, orthodontic intrusion of maxillary incisors can be a viable option as an alternative to surgical repositioning of maxilla.^12^ However, the true incisor intrusion is limited to 4 mm and its long term stability has not been demonstrated.^12-15^ The degree of upper incisor inclination is also related to upper incisor display, as retroclined incisors are usually more extruded.^7^

Variations in the upper lip length directly affect the MIDR.^16^ A short upper lip in relation to the underlying skeletal structures may result in an excessive MIDR and vice versa.^16^ ^,^ ^17^ In patients with short upper lip, if the surgical approach to increase the lip length is not opted, the potential of a successful orthodontic camouflage is reduced. However, patients with hyperactive lip elevator muscles may present with a normal MIDR but still show excessive gingival tissues during smile.^18^ Thus, along with the dental and skeletal components, the role of soft tissues in determining smile esthetics of an individual cannot be denied.

Only few studies addressed the association between these underlying skeletal, dental and soft tissue components and MIDR.^19^ ^,^ ^20^ Thus, the treatment of inappropriate display of maxillary incisors is usually limited to only few components that are easy to modify by orthodontic treatment or orthognathic surgery. The current study was designed to explore the role of different substructure attributes contributing to the display of maxillary incisors at rest, which may need to be altered by orthodontic or surgical treatment to improve dental esthetics. 

## MATERIAL AND METHODS

A retrospective cross-sectional study was conducted at The Aga Khan University Hospital, using the pretreatment orthodontic records of adult orthodontic patients aged 18 to 30 years. The sample size was calculated using the findings of Arriola-Guillen and Flores-Mir,^21^ who reported the correlation between the upper incisor display and upper lip height as -0.333. The power was set at 90% and alpha was kept as 0.05 to calculate the sample size, which showed a sample of 48 subjects was required. However, to increase the power of this study, the maximum number of available subjects was included. This resulted in a total sample of 150 subjects (75 males and 75 females). Ethical clearance was obtained from the ethical review committee of The Aga Khan University (ERC Exemption No. 4003-Sur-ERC-16) prior to the data collection.

Subjects with previous history of orthodontic treatment, trauma or surgery involving facial structures or with any craniofacial anomaly or syndrome were excluded from the study. 

The MIDR of all subjects was clinically measured using a millimeter scale, with the patient sitting upright, with lips completely relaxed. The maximum distance from the lowest point of upper lip to the incisal edge of any of the upper incisor was recorded as MIDR. The lateral cephalograms were recorded with the standardized method using Orthoralix 9200 (Gendex-KaVo, Milan, Italy). The technique involved rigid head fixation in a cephalostat and a 165-cm film-to-tube distance. The sagittal facial plane was held at a right angle to the path of the X-rays, while the Frankfort Horizontal Plane (FHP) of the subject was kept parallel to the horizontal plane. Teeth were occluded in the centric occlusion and lips were maintained in a relaxed position. 

**Figure 1:**
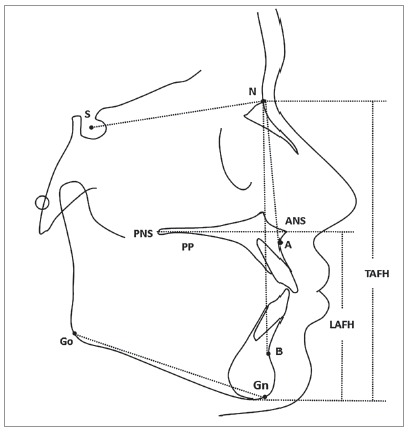
- Skeletal components: ANB angle, palatal plane angle, mandibular plane angle, lower anterior facial height (LAFH), total anterior facial height (TAFH). PP, palatal plane; ANS, anterior nasal spine; PNS, posterior nasal spine; Go, gonion; Gn, gnathion; N, nasion; S, sella; A, deepest point at the anterior aspect of maxillary alveolar process; B, deepest point at the anterior aspect of mandibular alveolar process.

**Figure 2:**
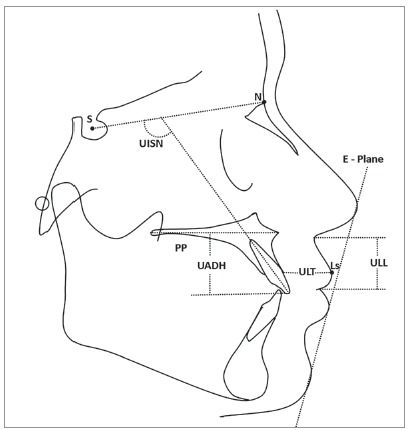
- Dental and soft tissue components: upper anterior dentoalveolar height (UADH); upper incisor to SN plane (UISN) angle; upper lip length (ULL); upper lip thickness (ULT); upper lip procumbency (the linear distance from Ls to the E line); PP, palatal plane; N, nasion; S, sella; E-plane, a plane joining the most prominent points of nose and chin; Ls, labrale superius - the most prominent point on the vermilion border of upper lip.

The lateral cephalograms of all the patients were manually traced by the main investigator on acetate paper, and the linear and angular measurements of all skeletal, dental and soft tissue components were performed with the help of a millimeter ruler and protractor, respectively (Figs 1 and 2). The following skeletal, dental and soft tissue components were included in the study: 

### Skeletal components

» ANB angle: angle formed by points A, N and B.
» Palatal plane angle: angle formed between SN plane and Palatal Plane (PP).
» Mandibular plane angle: angle formed between SN plane and GoGn plane.
» Lower anterior facial height (LAFH): linear distance from PP to Menton (Me).
» Total anterior facial height (TAFH): linear distance from nasion to Me.

### Dental components

» Upper incisor inclination (UISN): angle formed between the long axis of most prominent maxillary incisors and SN plane.
» Upper anterior dentoalveolar height (UADH): shortest distance from PP to the lowest point of maxillary incisor.

### Soft tissue components

» Upper lip length (ULL): linear distance from the junction of nasal columella and upper lip to the junction of upper and lower lips.
» Upper lip thickness (ULT): distance from labrale superius (Ls) to the alveolar bone crest in midline.
» Upper lip procumbency: shortest distance between E - plane and Ls, recorded as positive value if Ls is anterior to E - plane, and negative if Ls is posterior to E - plane.

To assess the measurement error, 30 lateral cephalograms were randomly selected and the steps of landmarks identification, tracing and measurement were repeated by the main researcher after three weeks of initial examination. Intra-class correlation coefficients were performed to assess the reliability for the two sets of measurements. The values of coefficients of reliability were found to be greater than 0.91 and 0.88 for all linear and angular variables, respectively.

Data were analyzed in SPSS for Windows (version 20.0, SPSS Inc. Chicago). Kolmogorov-Smirnov test was used to check the normality of the measurements. Independent sample t-test was used to compare the mean age and mean incisal display at rest, between males and females. Linear regression analyses were performed to assess the variations in maxillary incisal display explained by each component. A multiple linear regression model was generated based on the four strongest factors. A p-value < 0.05 was considered statistically significant.

## RESULTS

The mean age of males and females included in the study was comparable (p = 0.086). However, females presented a mean MIDR 1 mm greater than males (p = 0.011) (Table 1).

**Table 1:** Comparison of mean ages and maxillary incisor display at rest, between males and females.

	Males (n = 75) Mean ± SD	Females (n = 75) Mean ± SD	P - value
Age (years)	22.00 ± 4.13	22.21 ± 4.45	0.086
Incisal display at rest (mm)	3.72 ± 2.69	4.77 ± 2.24	0.011*

n = 150; SD = standard deviation; independent sample t-test.

* p < 0.05.

A simple linear regression analysis showed that several dental, skeletal and soft tissue components were significantly related to the MIDR (Table 2). The highest variances in MIDR were explained by upper lip length (29.7%), upper lip thickness (27.3%) and mandibular plane angle (25.8%). The palatal plane angle and total anterior facial height were least significantly associated with the MIDR, explaining only 0.06% and 0.00% variance, respectively. No significant association was found with age in the present study sample comprising the age group 18-30 years. 

**Table 2:** Simple linear regression analysis.

	Variable	r	P - value	Adjusted R^2^
Skeletal components	ANB Angle	0.311	<0.001*	9.1%
	Mandibular Plane Angle	0.513	<0.001*	25.8%
	Palatal Plane Angle	0.030	0.716	0.06%
	Lower Anterior Facial Height	0.341	<0.001*	11.0%
	Total Anterior Facial Height	0.079	0.336	0.00%
Dental components	Upper Incisor Inclination	-0.195	0.017*	3.2%
	Upper Anterior Dentoalveolar Height	0.169	0.039*	2.2%
Soft tissue components	Upper Lip Thickness	-0.527	<0.001*	27.3%
	Upper Lip Length	-0.549	<0.001*	29.7%
	Upper Lip Protrusion	0.207	0.011*	3.6%
Age		-0.047	0.629	0.00%

n = 150; Linear regression analysis.

* p < 0.05.

Multiple linear stepwise regression analysis was used to remove inter-correlation among the eight independent variables and to find out the clinically important variables that could predict the amount of MIDR. This resulted in a four-variable model incorporating mandibular plane angle, lower anterior facial height, upper lip thickness and upper lip length, explaining about of 63% variance in the MIDR (Table 3).

**Table 3:** Multiple linear regression model.

Variable	Coefficient (B)	Standard Error	P - value
Constant	4.816	1.568	0.003
Mandibular Plane Angle	0.094	0.025	<0.001*
Lower Anterior Facial Height	0.083	0.018	<0.001*
Upper Lip Thickness	-0.369	0.043	<0.001*
Upper Lip Length	-0.134	0.041	<0.001*

n = 150; Adjusted R^2^ = 0.634.

* p < 0.05.

## DISCUSSION

It is difficult to develop an accurate and reproducible method of assessing maxillary incisal display at smile that can be used universally.^23^ Several factors such as age, sex, emotional status, and circadian rhythms can affect the MIDR and the activity of the orofacial muscles involved in the dynamic process of smiling.^23^ ^-^ ^25^ All of these factors could not be controlled in the present study. A large sample size of only young adults with equal representation of males and females might have mitigated the effects of some confounders. Moreover, maxillary incisal display during other facial expressions and normal conversation is difficult to be objectively assessed. In this regard, MIDR has been found to be strongly correlated to the maxillary incisal display during function, and have been used to represent the dental component of the facial esthetics.^26^

A reduction in the MIDR is a part of the normal aging process. To reduce the impact of age, only young adults aged 18-30 years were included in this study, allowing for better analysis of MIDR relationship with different anatomic variables. The current study reported a sexual dimorphism in MIDR, which was in disagreement with the findings of other studies.^26^ ^,^ ^27^ However, other studies^11,28^ have shown that women show more maxillary incisal display than men, which is in agreement with the present results. The structural differences in the facial soft and hard tissues between males and females may explain a greater MIDR in women than men. An ultrasound-based investigation has shown that females have relatively thicker zygomaticus major muscles as compared to males.^29^ Similarly, a Class II jaw relationship is more frequently found in females, which is strongly correlated to a greater MIDR.^19^ However, larger size of clinical crowns in males may partially negate the effect of variations in soft tissue anatomy.^11^ Thus, interaction between several underlying components play a role in determining the ultimate proportion of maxillary incisors visible during rest and function. Interestingly, when several variables were considered in a multiple linear regression model, the gender failed to contribute significantly to the total variation in MIDR.

The present findings present upper lip length as the major etiological factor affecting maxillary incisal display. However, there are controversial reports about the role of upper lip length in the published literature. Some studies^13^ ^,^ ^16^ provide evidence that short upper lip is associated with excessive upper incisal display; whereas, other studies^18^ ^,^ ^27^ ^,^ ^30^ claim that a short upper lip is most frequently found in patients with short facial height and reduced incisal display. Despite these conflicting reports, orthodontists frequently consider a short upper lip as the cause of gummy smile. Surgical lip lengthening and use of Botox injections remain the main treatment for short upper lip.^31^ However, due to the invasive nature, unpredictable results and possible complications of surgical lip lengthening, and temporary results of Botox injections, most of the patients with gummy smile are treated with orthodontic intrusion of upper incisors, crown lengthening procedures or Le Fort I maxillary impaction.^31^ ^,^ ^32^

The morphological variation of maxilla, its rotation around the transverse axis and its position in sagittal plane, all have been implicated in the cases of an excessive or reduced MIDR. Anterior maxillary dentoalveolar height, also regarded as anterior maxillary height or vertical maxillary height in literature,^21^ have been shown to be significantly associated with the excessive incisor display.^9-11^ The morphology of anterior maxilla is determined by both genetic and environmental factors. Studies have shown that the upper anterior dentoalveolar height is affected by dental intrusion or extrusion, under the influence of different environmental or therapeutic factors; thus it was included in the dental components in the current study.^12^ ^,^ ^21^ Similarly, a clockwise rotation of maxillary base may result in an excessive MIDR, while a counter-clockwise rotation results in reduced incisal display.^11^ Lastly, the maxillary prognathism has been shown to be associated with an excessive maxillary incisal display.^19^ The current study investigated the role of anterior maxillary dentoalveolar height, the palatal plane angle and maxillary prognathism in relation to mandible in determining the amount of MIDR. No significant association was found between palatal plane angle and MIDR, while a weak positive correlation was found between anterior maxillary dentoalveolar height and MIDR. However, the maxillary position with respect to mandibular sagittal plane as assessed through ANB angle was significantly associated with the MIDR, explaining about 9% variance. These results are in agreement with the findings of previous studies.^19,20^ In addition, a Class II jaw relationship with maxillary prognathism is associated with a thin upper lip.^34^ A moderate negative correlation between the upper lip thickness and MIDR, as discovered in this study, explains the interaction between the skeletal and soft tissue components and its effect on MIDR.

Apart from the lip characteristics, the second factor that has most consistently been linked to MIDR is the vertical facial dimension.^34^ ^,^ ^35^ The vertical facial proportions are assessed by parameters such as total anterior facial height, lower anterior facial height, cranial base to mandibular plane angle, and Frankfort horizontal plane to mandibular plane angle. The current study contemplates cranial base to mandibular plane angle among the strongest predictors of MIDR, explaining about 25% of variance in MIDR. Similarly, lower anterior facial height was also found to be significantly associated with MIDR. A multitude of studies corroborate the present findings.^19^ ^,^ ^20^ The relevance of use of vertical pull headgear in growing children and surgical correction of increased facial dimension with Le Fort I maxillary impaction cannot be overemphasized in this regard.

Among dental components, Sabri^36^ claimed that proclination of maxillary incisors can significantly reduce MIDR. This might be true for some patients, however, upper incisor to SN plane inclination was not found to be significantly associated with MIDR in the current study. Similar findings were reported by Suh et al^20^ not only for upper incisor inclination, but also for other dental components such as overjet and overbite. Thus, the chief determinants of maxillary incisor display are soft and hard tissue components, and treatment should ideally be directed towards correction of these attributes. 

This analysis describes the association between the MIDR and different dental, skeletal and soft tissue components and provides insights of the etiological bases of inappropriate display of maxillary incisors. Findings of the current study may facilitate the decision-making process in orthodontic patients lacking an ideal maxillary incisal display, thus can help in making more efficient treatment plans for these patients. The orthodontic clinician can focus on the main underlying component, design an individualized treatment plan, and tailor a suitable mechanotherapy protocol according to the patient’s need. However, the variables included in the multiple linear regression model explain only 63% of the variation in MIDR, which indicates that other factors remain to be identified. 

The other limitation of the current study is the use of MIDR as the predictor of maxillary incisal display during function. In social circumstances, the maxillary incisal display during conversation, smile and other facial expressions has more practical significance, and thus should be analyzed accordingly. Hyperactivity of lip muscles has been reported as the possible cause of gummy smile by different researchers, and poor correlation has been reported between the MIDR and maxillary incisal display during smile in these patients.^16^ ^-^ ^18^ Thus, studies with methodology involving evaluation of smile dynamic could provide better explanations of etiological factors of unaesthetic display of maxillary incisors during function. 

## CONCLUSIONS

Maxillary incisal display at rest was generally greater in females than males. Upper lip length was found to be the strongest predictor of the maxillary incisal display at rest; however, several soft tissue, hard tissue and dental components affected MIDR. About two-third variance in the maxillary incisal display at rest was explained by the vertical facial pattern, and upper lip length and thickness.
